# Interleukin-1 receptor antagonist deficiency with a novel mutation; late onset and successful treatment with canakinumab: a case report

**DOI:** 10.1186/s13256-015-0618-4

**Published:** 2015-06-23

**Authors:** Ezgi Ulusoy, Neslihan Edeer Karaca, Hatem El-Shanti, Erhan Kilicoglu, Guzide Aksu, Necil Kutukculer

**Affiliations:** 1 Department of Pediatrics, Ege University Faculty of Medicine, Izmir, Turkey; 2 Qatar Biomedical Research Institute, Medical Genetics Center, Doha, Qatar

**Keywords:** Autoinflammation, Canakinumab, Interleukin-1 receptor antagonist deficiency

## Abstract

**Introduction:**

Interleukin-1 receptor antagonist deficiency is a rare autoinflammatory disease involving neonatal onset of pustulosis, periostitis, and sterile osteomyelitis. The underlying genetic abnormality involves a recessive mutation in *IL1RN*, which encodes interleukin-1 receptor antagonist. In this case report, we describe a case of a 12-year-old Turkish girl who initially was presented at 1 year of age, older than previously reported children with interleukin-1 receptor antagonist deficiency, and with a novel mutation, p.R26X, in *ILR1N*.

**Case presentation:**

Our patient developed pustular cutaneous lesions at 1 year of age. At the age of 12 years, she was hospitalized for arthralgia of her knees, elbows, and ankles and arthritis of the left knee, with simultaneous pustular cutaneous lesions. She was admitted to the intensive care unit because of septicemia and respiratory insufficiency during follow-up. A skin biopsy of hyperpigmented lesions demonstrated neutrophil infiltration in the epidermis and subepidermal pustular dermatosis. Interleukin-1 receptor antagonist deficiency was suspected, and genetic analysis revealed a homozygous mutation (p.R26X) in *IL1RN*, which led to a diagnosis of interleukin-1 receptor antagonist deficiency. Treatment with canakinumab (recombinant human anti-human interleukin-1β monoclonal antibody) 150mg subcutaneously once every 6 weeks was initiated. Our patient did not experience further cutaneous lesions or arthritis. Her post-treatment inflammatory markers were normal; she gained weight; and she was able to walk independently.

**Conclusions:**

In this case report, we describe a patient with interleukin-1 receptor antagonist deficiency who responded excellently to canakinumab treatment. We believe more awareness is warranted for interleukin-1 receptor antagonist deficiency in children. It is possible that the mutation in our patient was a founder mutation that may lead to diagnosis of additional cases in Turkey.

## Introduction

Hereditary autoinflammatory diseases (AID) are a heterogeneous group of rare genetic disorders characterized by recurrent episodes of inflammatory lesions that can affect the skin, joints, bones, eyes, gastrointestinal tract, and central nervous system (CNS), in association with signs of systemic inflammation without high-titer autoantibodies or antigen-specific T cells [1]. These diseases are caused by dysregulated activation of inflammasomes, which are critical for the activation of the proinflammatory cytokine interleukin (IL)-1β, a powerful mediator of inflammatory responses [2–6].

Deficiency of interleukin-1 receptor antagonist (DIRA) is a rare AID involving neonatal onset of pustulosis, periostitis, and sterile osteomyelitis [7, 8]. This disease shares some clinical features with neonatal onset multi-system inflammatory disease (NOMID)—signs of systemic inflammation and rash, beginning from birth—but it has a more severe clinical course and clinical features that are not observed in NOMID. These include severe osteopenia, lytic bone lesions, respiratory involvement, and thrombotic episodes [9, 10]. The underlying genetic abnormality involves a recessive mutation in *IL1RN*, which encodes for the interleukin-1 receptor antagonist (IL-1Ra) [1, 2, 11]. The radiological manifestations of DIRA syndrome include multi-focal osteitis of the ribs and long bones, heterotopic ossification, and periarticular soft tissue swelling [10]. Treatment with anakinra leads to a rapid clinical improvement [1, 2, 7–9, 11–13].

In this report, we describe a case of a 12-year-old girl who was initially presented at 1 year of age. She is considered to have a late-onset presentation in comparison to previously reported children with DIRA. She has a novel mutation in *IL1RN* and, to the best of our knowledge, is the first reported patient with DIRA who has had an excellent response to canakinumab (recombinant human anti-human IL-1β monoclonal antibody) treatment.

## Case presentation

A 12-year-old Turkish girl, born at 38 weeks of gestational age to unrelated healthy parents, was well until 1 year of age, when she developed pustular cutaneous lesions that responded to corticosteroid and antibiotic treatment with healing and scar formation. Various treatments of these lesions had required four hospitalizations during the previous 11 years. No other family member had similar skin conditions. She was hospitalized at the age of 12 years for arthralgia of her knees, elbows, and ankles and arthritis of her left knee, with concomitant pustular cutaneous lesions. She developed septicemia and was admitted to the intensive care unit of a public hospital with respiratory insufficiency during her follow-up. After recovery, she was referred to our pediatric immunology department for further evaluation.

**Fig. 1:**
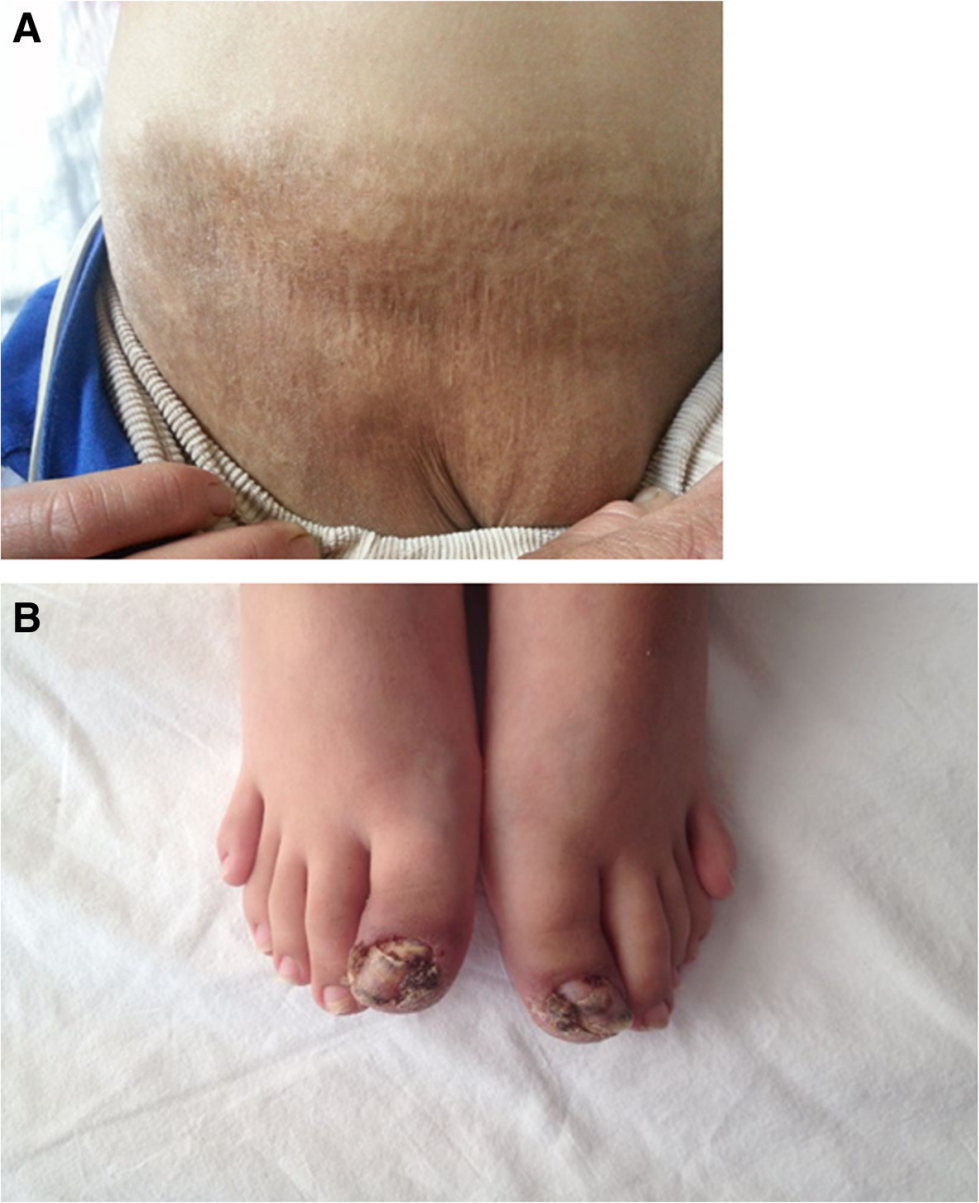
**a** Inguinal and pubic hyperpigmented scar lesions. **b** Paronychia of the toes

A hyperpigmented scar lesion on the right side of the face; bilateral inguinal, paraumbilical hyperpigmented scar lesions; and paronychia of the thumbs were noted on admission (Fig. 1). Additionally, the patient had contracture of the left knee limiting her motion, episcleritis, and failure to thrive [25kg (below third percentile), 132cm (below third percentile)]. The results of her laboratory studies revealed iron deficiency anemia, hypergammaglobulinemia, and elevated acute-phase reactants (red blood cell count 4.2 million/mm^3^, hemoglobin 9.1g/dl, hematocrit 27.8%, mean corpuscular volume 81fl, thrombocytes 254,000/mm^3^, immunoglobulin G (IgG) 1760mg/dl, IgM 186mg/dl, IgA 195mg/dl, C-reactive protein 6.5mg/dl, erythrocyte sedimentation rate 100mm/hr, and serum amyloid A 123mg/L). Total IgE level, eosinophil count, lymphocyte subset levels, and the oxidative burst activity of granulocytes were normal. Autoantibodies (anti-nuclear antibody, anti-neutrophilic cytoplasmic antibody, and rheumatic factor) were negative. She was evaluated for tuberculosis and was found to have two bacillus Calmette-Guérin scars, a 12mm tuberculin response, and a negative QuantiFERON® assay result (QIAGEN, Chadstone, Australia). Serologic investigations yielded negative results for cytomegalovirus, Epstein-Barr virus, hepatitis B and C viruses, syphilis, and HIV. Her blood and urine cultures were negative for bacteria. Splenomegaly was detected by abdominal ultrasonography. Skin biopsy of hyperpigmented lesions demonstrated neutrophil infiltration in epidermis and subepidermal pustular dermatosis. The results of chest radiography and skeletal surveys were normal.

DIRA was clinically suspected on the basis of clinical similarities between our patient and other patients with DIRA described in the literature to date. The resequencing of the entire coding sequence of *IL1RN* and the flanking splice sites revealed a homozygous mutation (p.R26X) confirming DIRA. Treatment with canakinumab 150mg subcutaneously once every 6 weeks was initiated, and a full response was achieved. She did not experience any cutaneous lesions or arthritis during 12 months of treatment and follow-up. Her inflammatory markers regressed to normal values (Table 1). She was able to walk independently, and gradual weight gain was observed. Treatment-related adverse events were not detected.

**Table 1:** Acute-phase reactants upon admission and during follow-up while receiving canakinumab treatment

	Reference ranges	On admission	After first administration of canakinumab	Last visit
CRP	0-0.1mg/dl	6.5mg/dl	1mg/dl	<0.33mg/dl
ESR	0-20mm/hr	100mm/hr	30mm/hr	10mm/hr
SAA	0-8mg/L	123mg/L	13.3mg/L	6.9mg/L

*CRP* C-reactive protein, *ESR* erythrocyte sedimentation rate, *SAA* Serum amyloid A

## Discussion

We describe a patient with DIRA with a homozygous mutation in *IL1RN.* AID can present with different skin lesions. The most common AID with pustular skin lesions are early onset inflammatory bowel diseases, Majeed syndrome, PAPA syndrome (pyogenic arthritis, pyoderma gangrenosum and acne), IL-36 antagonist deficiency, *CARD14*-mediated psoriasis, and DIRA [14]. DIRA [OMIM:612852] was first described in 2009 [1]. It is an autosomal recessive AID in which absence of IL-1Ra allows unopposed IL-1 activation. An increased response to IL-1α and IL-1β stimulation results in life-threatening systemic inflammation with prominent skin and bone involvement [1, 15]. DIRA is a rare condition that usually presents in the neonatal period. Patients with DIRA present with systemic inflammation, respiratory distress, joint swelling, pustular rash, multi-focal osteomyelitis, and periostitis typically affecting distal ribs and long bones [1–3, 11–15]. Our patient had systemic inflammation, joint pain, and pustular lesions that healed with scars resembling those of previously reported patients with DIRA. However, she did not have typical periostitis with osteolysis. Rare vascular manifestations, such as thrombosis of the right common femoral and right external iliac veins and CNS vasculitis, have been described in three DIRA patients [1, 9, 15]. Our patient had no signs of vascular involvement. The clinical diagnosis of DIRA was supported by the skin biopsy showing neutrophil infiltration of the epidermis and subepidermal pustular dermatosis.

Symptom onset in our patient occurred at 1 year of age, which was late in comparison with previously reported patients with DIRA. These patients were presented for treatment within 1 month of birth [1, 7, 9]. Sakran and colleagues [16] reported a case of a girl from Israel who was symptomatic at the age of 4 months. They emphasized later symptom onset of their patient compared with previously reported patients. Our patient had no pustular lesions in infancy and no joint pain until she was 12 years old. To the best of our knowledge, she is the first reported patient with DIRA in the literature with a healthy neonatal period. Genetic analysis revealed a novel *IL1RN* mutation, p.R26X, confirming the clinical diagnosis of DIRA.

To date, patients with DIRA in Newfoundland, the Netherlands, Lebanon, Puerto Rico, Brazil and Israel have been described [1, 8, 9, 12–16]. In addition to those patients, Altınok and colleagues [2] reported two siblings in Turkey in 2012 with a novel mutation, Q119X. Our patient is the third patient with DIRA in Turkey reported to date, and the only one of the three who is still alive.

Conventional disease-modifying anti-rheumatic drugs, including steroids, are of limited benefit, but specific IL-1-targeting therapies dramatically improve clinical symptoms within days, normalize acute-phase reactants, and permit appropriate growth. So far, three IL-1 blockers have been approved: anakinra, rilonacept, and canakinumab [16]. Anakinra, a recombinant human IL-1Ra that blocks the proinflammatory effects of IL-1-β, rapidly relieves the symptoms of systemic inflammation in patients with DIRA [1, 17]. Anakinra is usually given subcutaneously at an initial dose of 1mg/kg/day. Most of the patients with DIRA are reported to have a good response to anakinra treatment. Unfortunately, our patient lived in a rural area where application of daily subcutaneous injections is unavailable, owing to inadequate sanitary conditions. Thus, canakinumab, a human anti-IL-1β monoclonal antibody that can be administered every 6 to 8 weeks, was the treatment of choice. We obtained full clinical and laboratory remission with canakinumab treatment applied every 6 weeks. To the best of our knowledge, ours is the first patient with DIRA treated with canakinumab.

## Conclusions

Patients with DIRA may present with systemic inflammation, respiratory distress, joint swelling, pustular rash, multi-focal osteomyelitis, and periostitis. DIRA must be considered in the differential diagnosis of children with these symptoms. Treatment with IL-1-β blockage is accepted to be lifesaving, because the disorder mimics severe bacterial infections and may result in death from development of systemic inflammatory response. A prompt and accurate diagnosis is of utmost importance to avoid improper management of patients with antibiotics alone, as effective treatment options are readily available. Our aim in this report is to raise awareness of the diagnosis of DIRA and adequate treatment choices for achieving remission and preventing permanent damage and early mortality, leading to improved quality of life in these patients.

## Consent

Written informed consent was obtained from the patient’s parents for publication of this case report and any accompanying images. A copy of the written consent is available for review by the Editor-in-Chief of this journal.

## Abbreviations

AID: autoinflammatory disease
CNS: central nervous system
CRP: C-reactive protein
DIRA: deficiency of interleukin-1 receptor antagonist
ESR: erythrocyte sedimentation rate
IgA: immunoglobulin A
IgG: immunoglobulin G
IgM: immunoglobulin M
IL: interleukin
IL-1Ra: interleukin-1 receptor antagonist
NOMID: neonatal onset multi-system inflammatory disease
SAA: serum amyloid A

## References

[1] Aksentijevich I, Masters SL, Ferguson PJ, Dancey P, Frenkel J, van Royen-Kerkhoff A, et al. An autoinflammatory disease with deficiency of the interleukin-1-receptor antagonist. N Engl J Med. 2009;360(23):2426–37. 10.1056/NEJMoa0807865 PMC2876877 19494218

[2] Altıok E, Aksoy F, Perk Y, Taylan F, Kim PW, Ilıkkan B, et al. A novel mutation in the interleukin-1 receptor antagonist associated with intrauterine disease onset. Clin Immunol. 2012;145(1):77–81. 10.1016/j.clim.2012.08.003 PMC3463868 22940634

[3] Gabay C, Lamacchia C, Palmer G. IL-1 pathways in inflammation and human diseases. Nat Rev Rheumatol. 2010;6(4):232–41. 10.1038/nrrheum.2010.4 20177398

[4] Hoffman HM, Wanderer AA. Inflammasome and IL-1β-mediated disorders. Curr Allergy Asthma Rep. 2010;10:229–35. 10.1007/s11882-010-0109-z PMC2892083 20425006

[5] Gross O, Thomas EJ, Guarda G, Tschopp J. The inflammasome: an integrated view. Immunol Rev. 2011;243(1):136–51. 10.1111/j.1600-065X.2011.01046.x 21884173

[6] Goldbach-Mansky R. Immunology in clinic review series; focus on autoinflammatory diseases: update on monogenic autoinflammatory diseases: the role of interleukin (IL)-1 and an emerging role for cytokines beyond IL-1. Clin Exp Immunol. 2012;167(3):391–404. 10.1111/j.1365-2249.2011.04533.x PMC3374271 22288582

[7] Stenerson M, Dufendach K, Aksentijevich I, Brady J, Austin J, Reed AM. The first reported case of compound heterozygous *IL1RN* mutations causing deficiency of the interleukin-1 receptor antagonist. Arthritis Rheum. 2011;63(12):4018–22. 10.1002/art.30565 21792839

[8] Minkis K, Aksentijevich I, Goldbach-Mansky R, Magro C, Scott R, Davis JG, et al. Interleukin 1 receptor antagonist deficiency presenting as ınfantile pustulosis mimicking ınfantile pustular psoriasis. Arch Dermatol. 2012;148(6):747–52. 10.1001/archdermatol.2011.3208 PMC3474848 22431714

[9] Reddy S, Jia S, Geoffrey R, Lorier R, Suchi M, Broeckel U, et al. An autoinflammatory disease due to homozygous deletion of the *IL1RN* locus. N Engl J Med. 2009;360(23):2438–44. 10.1056/NEJMoa0809568 PMC2803085 19494219

[10] Aksentijevich I, Nowak M, Mallah M, Chae JJ, Watford WT, Hofmann SR, et al. De novo *CIAS1* mutations, cytokine activation, and evidence for genetic heterogeneity in patients with neonatal-onset multisystem inflammatory disease (NOMID): a new member of the expanding family of pyrin-associated autoinflammatory diseases. Arthritis Rheum. 2002;46(12):3340–8. 10.1002/art.10688 PMC4556432 12483741

[11] Thacker PG, Binkovitz LA, Thomas KB. Deficiency of interleukin-1-receptor antagonist syndrome: a rare auto-inflammatory condition that mimics multiple classic radiographic findings. Pediatr Radiol. 2012;42(4):495–8. 10.1007/s00247-011-2208-y 21789664

[12] Jesus AA, Osman M, Silva CA, Kim PW, Pham TH, Gadina M, et al. A novel mutation of *IL1RN* in the deficiency of interleukin-1 receptor antagonist syndrome: description of two unrelated cases from Brazil. Arthritis Rheum. 2011;63(12):4007–17. 10.1002/art.30588 PMC3463867 22127713

[13] Schnellbacher C, Ciocca G, Menendez R, AksentijevichI I, Goldbach-Mansky R, Duarte AM, et al. Deficiency of ınterleukin-1 receptor antagonist responsive to anakinra. Pediatr Dermatol. 2013;30(6):758–60. 10.1111/j.1525-1470.2012.01725.x PMC3548019 22471702

[14] Almeida de Jesus A, Goldbach-Mansky R. Monogenic autoinflammatory diseases: concept and clinical manifestations. Clin Immunol. 2013;14(3):155–74. 10.1016/j.clim.2013.03.016 PMC3769788 23711932

[15] Brau-Javier CN, Gonzales-Chavez J, Toro JR. Chronic cutaneous pustulosis due to a 175-kb deletion on chromosome 2q13: excellent response to anakinra. Arch Dermatol. 2012;148(3):301–4. 10.1001/archdermatol.2011.2857 PMC3313085 22431772

[16] Sakran W, Shalev SA, El-Shanti H, Uziel Y. Chronic recurrent multifocal osteomyelitis and deficiency of interleukin-1-receptor antagonist. Pediatr Infect Dis J. 2013;32(1):94. 10.1097/INF.0b013e3182700cc1 23241992

[17] Dinarello CA, van der Meer JW. Treating inflammation by blocking interleukin-1 in humans. Semin Immunol. 2013;25(6):469–84. 10.1016/j.smim.2013.10.008 PMC3953875 24275598

